# Alzheimer's Disease Detection Through Whole-Brain 3D-CNN MRI

**DOI:** 10.3389/fbioe.2020.534592

**Published:** 2020-10-30

**Authors:** Guilherme Folego, Marina Weiler, Raphael F. Casseb, Ramon Pires, Anderson Rocha

**Affiliations:** ^1^Institute of Computing, University of Campinas, Campinas, Brazil; ^2^CPQD, Campinas, Brazil; ^3^Laboratory of Behavioral Neuroscience, National Institute on Aging, National Institutes of Health, Intramural Research Program (NIA/NIH/IRP), Baltimore, MD, United States; ^4^Seaman Family MR Research Center, Cumming School of Medicine, University of Calgary, Calgary, AB, Canada

**Keywords:** Alzheimer's disease, computer aided diagnosis, artificial intelligence, computer vision, deep learning, convolutional neural networks, image classification, magnetic resonance imaging

## Abstract

The projected burden of dementia by Alzheimer's disease (AD) represents a looming healthcare crisis as the population of most countries grows older. Although there is currently no cure, it is possible to treat symptoms of dementia. Early diagnosis is paramount to the development and success of interventions, and neuroimaging represents one of the most promising areas for early detection of AD. We aimed to deploy advanced deep learning methods to determine whether they can extract useful AD biomarkers from structural magnetic resonance imaging (sMRI) and classify brain images into AD, mild cognitive impairment (MCI), and cognitively normal (CN) groups. We tailored and trained Convolutional Neural Networks (CNNs) on sMRIs of the brain from datasets available in online databases. Our proposed method, ADNet, was evaluated on the CADDementia challenge and outperformed several approaches in the prior art. The method's configuration with machine-learning domain adaptation, ADNet-DA, reached 52.3% accuracy. Contributions of our study include devising a deep learning system that is entirely automatic and comparatively fast, presenting competitive results without using any patient's domain-specific knowledge about the disease. We were able to implement an end-to-end CNN system to classify subjects into AD, MCI, or CN groups, reflecting the identification of distinctive elements in brain images. In this context, our system represents a promising tool in finding biomarkers to help with the diagnosis of AD and, eventually, many other diseases.

## 1. Introduction

Dementia by Alzheimer's disease (AD) is characterized by multiple cognitive problems, including difficulties in memory, executive functions, language, and visuospatial skills. The most significant risk for AD is aging—there is almost a 15-fold increase in the prevalence of dementia between the ages of 60 and 85 years (Evans et al., 1989). The projected burden of the disease represents a looming healthcare crisis as the population of most industrialized countries continues to grow older. Although there is still no cure, it is possible to treat both cognitive and behavioral symptoms of AD.

The early diagnosis of the disease is paramount for interventions, and clinical trials in AD tend to enroll subjects at earlier time-points before neuronal degeneration has achieved a particular stage and treatment is often more effective. In this context, neuroimaging is one of the most promising areas of research for early detection of AD, as the progressive degeneration of brain structures can be seen as a dramatic cerebral shrinkage in structural magnetic resonance imaging (sMRI).

Thus far, works in this area have recurrently considered only a small number of subjects and images, often with curated data (i.e., reviewed, prepared, and organized by experts), such as ADNI's Standardized MRI Data Sets (Wyman et al., 2013). Additionally, with the lack of a standard evaluation protocol, each study employed its criteria, with its own random data split. The lack of standardization limits the comparison between different methods and usually overestimates performance in a real-world scenario. When data is not readily preprocessed and comes from different sources, this situation is even more problematic.

A recent and extensive review (Wen et al., 2020) indicated that a reasonable number of studies using convolutional neural networks (CNNs) for AD either present evident data leakage problems, or offer scarce explanation for the validation method to ensure that data leakage has not occurred. Data leakage possibilities only emphasize the need for an independent set of images for evaluation. In addition to this review, Wen et al. (2020) proposed a standard framework for rigorous performance assessment, using data from ADNI, AIBL (Ellis et al., 2009), and OASIS (Marcus et al., 2007, 2009).

For fair comparisons between different methods, a few challenges with standard protocols and hidden test labels were launched, such as the CADDementia challenge (Bron et al., 2015). Although presenting a good classifier—63.0% accuracy in classifying MRI images into cognitively normal (CN), mild cognitive impairment (MCI), and AD patients—, the winning method (Sørensen et al., 2017) used transductive inference to calculate hippocampal shape scores, requiring the CADDementia test data to be calculated, which deviates from the original proposal of applying the algorithm in the clinical setting. Additionally, their pipeline failed to process three scans from the CADDementia test set, requiring manual intervention. The analysis of each subject took 19 h of computation time. The second-best team (Wachinger and Reuter, 2016) employed a domain-adaptation approach, and optimization was done on the union of ADNI and CADDementia training sets, with equal weights for each sample. The analysis of each subject took 17.4 h of computation time.

Among the available machine learning methods, CNNs have been increasingly used in the Alzheimer's biomarker identification task, given its power to learn discriminative representations hierarchically in an automated fashion. Most studies employing CNNs in the context of AD used 2D inputs, whereas studies that used 3D inputs focused basically on binary classifications. The few works that trained a 3D CNN for the multiclass CN/MCI/AD classification evaluated their performances with cross-validation on ADNI data and considered only networks comparable to our smallest proposed architecture. All of this context highlights the novelty in our research, as we optimized very deep 3D CNNs, with up to 22 layers, for the multiclass diagnosis task, and evaluated our performance on the CADDementia challenge, with unknown labels, making our results much more reliably applicable in a real-world setting.

A previous work that employed the deep learning approach in the context of AD (Korolev et al., 2017) designed 3D CNNs based on smaller versions of VGG (Simonyan and Zisserman, 2014) and ResNet (He et al., 2016) architectures. However, only binary classification tasks were considered, which were evaluated using cross-validation on ADNI. The first group to successfully propose a deep-learning approach to the CADDementia challenge (Dolph et al., 2017) extracted features such as cortical thickness, surface area, volumetric measurements, and texture. These values were used to greedily layer-wise train a stacked auto-encoder with three hidden layers, achieving competitive results. Using the whole brain as input, Hosseini-Asl et al. (2018) employed a small stack of unsupervised 3D convolutional auto-encoders (3D-CAE), evaluating with cross-validation on ADNI. One of the works more closely related to ours (Esmaeilzadeh et al., 2018) optimized small 3D CNNs, similar to our most basic model, and considered the multiclass classification task. However, the performance was measured using ADNI cross-validation, hindering better comparisons with our method. It is worth noting that they reported a classification accuracy of 61.1%, but observed overfitting in training data.

In more recent work, Abrol et al. (2020) developed 3D CNNs based on the ResNet architecture and experimented with several binary and multiclass tasks. They used the ADNI data to create a training set, used for cross-validation, and a small test set. Even though their results were promising, no further comparisons with different datasets or standardized evaluation frameworks were made. Interestingly, their experiments also presented overfitting in training data. Also recently, Mehmood et al. (2020) adapted the VGG architecture to create a 2D siamese CNN. They evaluated their model using training and test split in the OASIS dataset, presenting compelling results. However, given their proposed data flow chart, it is possible that their data augmentation technique also introduced data leakage.

In our research, we relied upon a 3D CNN with data primarily provided by ADNI (Mueller et al., 2005) and evaluated on the CADDementia challenge (Bron et al., 2015). Our solution also includes an accountable mechanism to allow us to understand its decisions. Our experiments were conducted in a scenario similar to real-world conditions, in which a CAD system is used on a dataset that is different from the one used for training.

The main challenges and contributions of our research included devising a deep-learning solution completely automatic and comparatively fast, while also presenting competitive results without using any domain-specific knowledge. Our method, named ADNet, yields considerable gains in accuracy, outperforming several other systems in the prior art, all of which require prior knowledge of the disease, such as specific regions of interest from input images. Alternatively, our system does not require any manual intervention, clinical information, or *a priori* selected brain regions. The main reason for not using any information from the disease is to empower the system to automatically learn and extract relevant patterns from regions of the brain, and eventually enable it to support current diagnosis standards for known or new diseases. In addition, it runs 80 times faster than the state of the art (Sørensen et al., 2017), on average.

Our generated ADNet and ADNet-DA models, as well as supporting code, are publicly available^1^ to be used or trained on new data. With this work, we are releasing one of the first models ready to use, encouraging open science and reproducible research, while also setting a starting point for researchers working with 3D MRIs.

## 2. Methodology

**Figure 1:**
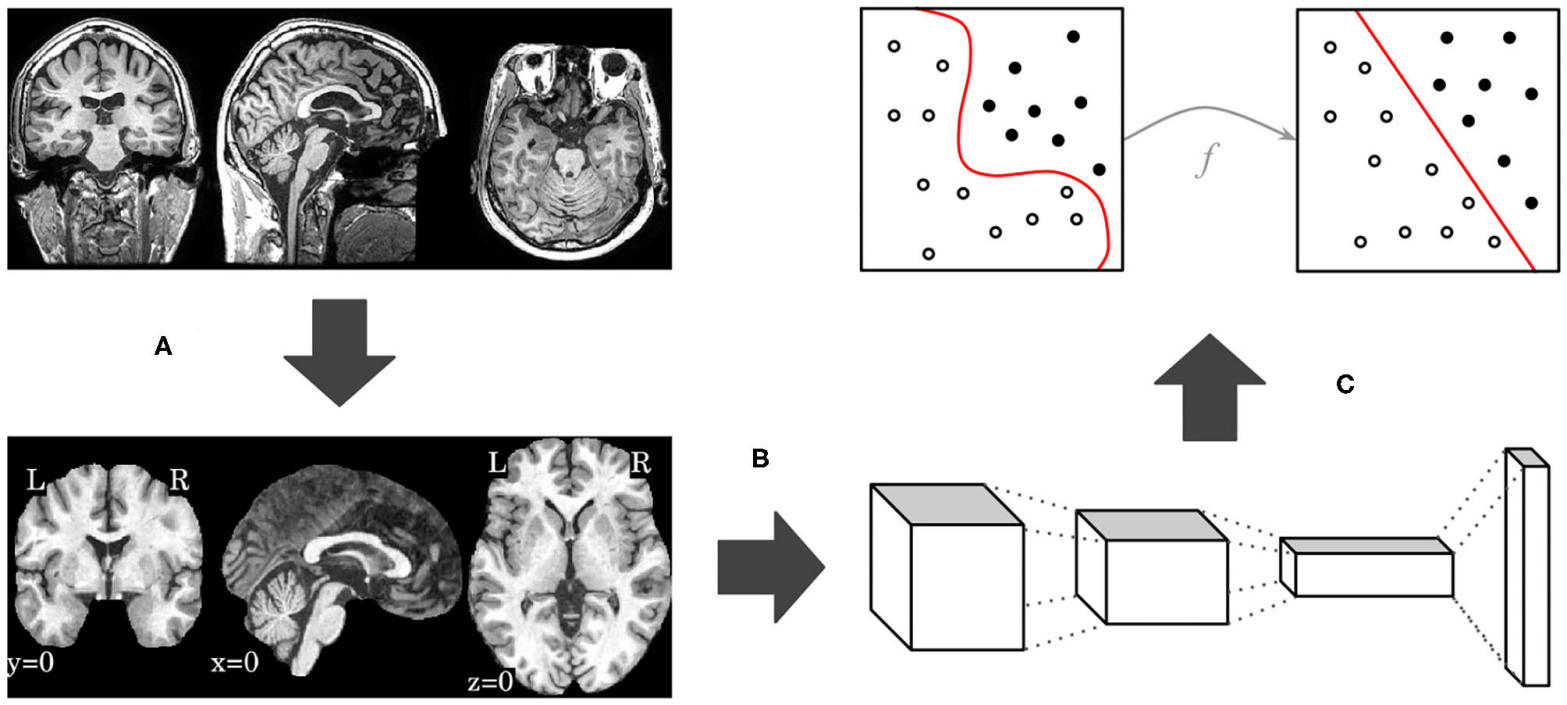
Overview of our proposed pipeline, with brain extraction and normalization **(A)**, 3D CNN processing **(B)**, and domain adaptation **(C)** steps, in this order.

In this work, we propose an end-to-end deep 3D CNN for the multiclass AD biomarker identification task, using the whole image volume as input. Our pipeline, displayed in Figure 1, is composed of three main steps: brain extraction and normalization (Figure 1A), 3D CNN processing (Figure 1B), and domain adaptation (Figure 1C). This section provides details of our pipeline, including image preprocessing, CNN architectures, and optimization techniques.

### 2.1. Brain Extraction and Normalization

Optimizing deep-learning systems using sMRIs in their original space requires the systems to learn discriminative patterns invariant to several transformations, demanding larger models and an even larger number of samples, with all expected variations. By registering our images to a standard template, we can expect similar structures to be roughly in the same spatial location, allowing us to handle the entire image at once and automatically determine the most important regions of interest.

We used the Advanced Normalization Tools (ANTs; Avants et al., 2009) to extract and normalize brain images. Our pipeline was based on previously defined scripts^2^ (Avants et al., 2011; Tustison and Avants, 2013), and we made use of the provided default parameters, including transformation types, sequence, and metrics. Essentially, our brain extraction and normalization pipeline comprised the following steps: image intensity winsorizing, bias field correction, another winsorizing step, translation alignment, rigid transform, affine transform, deformable symmetric normalization (SyN), application of brain mask from the atlas, and range normalization.

As we used registered brains in our research, we opted for a less rigid and less linear atlas, allowing for some degree of variation during the registration process. This atlas also had a high spatial resolution, so finer details would not be lost in the process. As such, the Montreal Neurological Institute (MNI) 152 International Consortium for Brain Mapping (ICBM) 2009c Nonlinear Asymmetric 1 × 1 × 1 *mm*^3^ (Collins et al., 1999; Fonov et al., 2009, 2011) atlas was chosen.

After the brain extraction and normalization process, the output image has the same dimensions as the atlas (i.e., 193 × 229 × 193). From all of these 8, 530, 021 voxels, only 1, 886, 574 (22%) of them are not zero. Since the brain is enclosed in a smaller region inside the image, we removed the border dimensions that contained no information, resulting in a final image of 145 × 182 × 155. This new space represents 48% of the original volume, reducing sparsity from 78 to 54%. Finally, we used the training set to compute mean and variance, then used them across all sets to normalize the data to zero mean and unit variance. Given that the used datasets did not fit in main memory, we adopted a single-pass online mean and estimated variance algorithm (Welford, 1962).

Our main challenge was putting together a registration pipeline, including the adopted atlas, that provided useful and meaningful results in a reasonable time, while maintaining high resolution images.

### 2.2. Convolutional Neural Network

We describe here the convolutional neural network architectures we adopted and the modifications we performed to each. These networks were initially designed for 2D color images, and we are dealing with 3D grayscale MRIs. Thus, the most natural adaptation was to convert all 2D operations, such as convolution or pooling, to 3D. Given these adaptations, we were unable to employ a transfer learning approach (Sharif Razavian et al., 2014) with the original networks directly.

A common attribute to all considered architectures is that spatial dimension is reduced as information flows to deeper layers. Spatial dimensionality reduction is usually achieved with max-pooling layers, or with more substantial strides in convolutional layers. To accommodate different data shapes that were not necessarily divisible by two, we adopted an *ad-hoc* approach by zero-padding each layer as needed, so no information was lost. We also added batch normalization (Ioffe and Szegedy, 2015) to every convolutional and fully-connected layers. All activation functions were rectified linear units (ReLU; Nair and Hinton, 2010), except for the classification output, which was a softmax function. Finally, the number of layers varied according to the adopted network standard. Table 1 shows the CNN architectures evaluated, taking into account the original approach for layer counting in each network.

**Table 1:** CNN architectures evaluated in this study.

**Architecture**	**Layers**	**Parameters (in millions)**
LeNet-5	7	0.3
VGG 2048	11	89.8
VGG 512	11	26.8
GoogLeNet	22	14.6
ResNet A	18	33.0
ResNet B	18	33.2

We started with a small network, based on the LeNet-5 architecture (LeCun et al., 1989; Lecun et al., 1998). Because this network is significantly older than the others, it required the largest modifications. This network was composed of the following layers: convolution, subsampling, convolution, subsampling, fully connected (originally implemented as convolutional), fully connected, and output. As subsampling layers had learnable parameters, we converted them to convolutions, with filter (kernel) size and stride equal to 2 × 2 × 2, thus keeping the subsampling behavior. The main difference was in the connection between the first subsampling and second convolutional layers, for which the particular arrangement in the original work was converted to a dropout layer with a probability of 40%. Similar to the original architecture, if we had adapted the last convolutional layer to match the previous layer's output size, it would have had 120 feature maps with a kernel size of 34 × 43 × 36, seriously increasing the number of parameters. To mitigate these issues, we adapted those kernels to 5 × 5 × 5 and added a global average pooling layer immediately following, similar to GoogLeNet (Szegedy et al., 2015) and ResNet (He et al., 2016). Naturally, the last layer contained three units (one for each class), with a softmax function activation.

The Visual Geometry Group (VGG) proposed deep CNN architectures, achieving second place in the classification task at ImageNet Large Scale Visual Recognition Challenge (ILSVRC) 2014 (Simonyan and Zisserman, 2014). They designed very uniform architectures ranging from 11 (configuration A) to 19 (configuration E) weight layers, i.e., considering only convolutional and fully-connected layers. Due to its uniformity, mostly with filters of size 3 × 3, the VGG architecture is considerably large. The first layers, with the original input dimensions, consumed a large GPU memory, while the last layers, with dense connections, generated several parameters. Since our input data were already quite large when compared to traditional 2D images, we adapted the VGG network configuration A by halving all numbers of filters in convolutional layers, and all numbers of units in fully-connected layers, while keeping filters sizes of 3 × 3 × 3 and dropout rate at 50%. Even after reducing the network size, the first fully-connected layer of our adapted VGG-A, with 2,048 units, accounted for 78, 643, 200 (88%) parameters. For comparison, Table 1 also includes our VGG-A with 512 units in the fully-connected layers.

While VGG achieved second place in ILSVRC-2014, GoogLeNet secured first place in the classification task (Szegedy et al., 2015), proposing an architecture named Inception. The basic idea was to increase both depth and width while keeping computational requirements constrained. This approach led to a deeper model with fewer parameters and better performance. We adapted directly from their GoogLeNet architecture, i.e., only discarding the local response normalization (Krizhevsky et al., 2012) layer and the auxiliary networks. We also adjusted the last average pooling layer, following the output shape of the previous layer, and kept the dropout rate at 40%. In this architecture, the number of layers came from depth, where single convolutional or fully-connected layers counted as one, while inception modules counted as two. However, each inception module had six individual internal convolutional layers.

In ILSVRC-2015, Residual Network (He et al., 2016) won first place in classification, localization, and detection tasks. These researchers wanted to understand whether learning better networks meant simply stacking more layers. With this study, they found the degradation problem, where traditional models similar to VGG stopped improving performance after a certain number of layers, and even started getting worse afterwards. To overcome this problem, they proposed the residual function, which is the basic building block of a Residual Network (ResNet). We adapted ResNet directly from the non-bottleneck 18-layer architecture, in which shortcuts with increasing dimensions were either (A) identity shortcuts, i.e., padding with zero, or (B) projection shortcuts, i.e., convolutions with 1 × 1 × 1 filter (kernel) size. Similarly to VGG, the number of layers came from convolutional and fully-connected layers, with projection convolutions not considered in the layer count.

In summary, we adopted four CNN architecture designs, namely, LeNet-5, VGG, GoogLeNet, and ResNet. LeNet-5 is considerably older and smaller, so it shall have a lower probability of overfitting. The VGG network is known for its uniformity, which makes it relatively simple to adapt, inspect, and use for many different tasks; however, this characteristic also makes it significant in the number of parameters and in hardware requirements. These drawbacks were addressed in both GoogLeNet and ResNet architectures, which also adopted very specific building blocks, making it possible to extract more complex patterns from data, while also increasing the number of layers and reducing the number of parameters. The idea was to explore different architectures and understand how they would behave in the AD task.

To avoid overfitting, we adopted regularization with L1 and L2 norms. In L1, this effect is achieved by minimizing the absolute values of the weights, while in L2 this is done with their squared values. In principle, L2 norm tends to produce diffuse and small numbers, while L1 tends to produce sparse numbers. This property makes L1 particularly well-suited to handle noisy data, acting as a feature selection algorithm, which could help us better visualize and explain what the CNN has learned. However, in general, L2 can be expected to provide superior results over L1.

All network architectures and their optimization were implemented using upstream (i.e., the latest version from the code repository) Lasagne (Dieleman et al., 2015), which is a deep learning framework based on Theano (Al-Rfou et al., 2016). At the time this research was carried out, we used a development version of Lasagne 0.2, and a development version of Theano 0.9.0, with Python 2.7.6, CUDA 7.5, and CuDNN 5. Additionally, we used SciKit-learn 0.18.1 (Pedregosa et al., 2011) and NumPy 1.11.3 (van der Walt et al., 2011).

### 2.3. Domain Adaptation

In addition to brain processing and CNN pipelines, we considered a domain adaptation approach. In our method, we trained a system using one dataset and evaluated it on a different dataset (i.e., CADDementia). Even though they are related, such differences mean that the source data distribution could be different from the target data distribution. Thus, it should be possible to further improve the results by adapting the previously-trained system to the new dataset, even if using only a small number of samples from this target domain. This scenario, also known as cross dataset validation, is more closely related to a real-world scenario, in which data sources will most likely be different between training and actual usage. Additionally, this is a more reliable way of assessing generalization capabilities of a machine learning algorithm.

In our domain adaptation approach, we started by using our previously-optimized CNN to extract features from the complete target dataset (i.e., CADDementia), in one of the last CNN layers. After, we normalized these features to zero mean and unit variance, using only the target training set to compute the parameters. With the normalized data, we optimized a one-vs.-rest logistic regression (McCullagh, 1984) on the complete target training set. In order to find the best parameters for this classifier, we used grid search with leave-one-out cross-validation. Then, we finally had a system that was enhanced for the target domain, making it possible to output improved classification probabilities for each sample in the target domain. This pipeline is similar to a transfer-learning approach (Sharif Razavian et al., 2014).

## 3. Experimental Setup

Given that training a CNN from scratch usually requires massive amounts of data, we gathered as many different imaging sources as possible. We collected an AD sMRI dataset comprising 23,165 images. Below, we describe our optimization approach, including associated parameters.

### 3.1. Data

In our data collection process, we considered the datasets indicated in Table 2. ADNI1 originally included three participant groups: CN, MCI, and AD. Starting in ADNIGO, the MCI stage was split into early MCI (eMCI) and late MCI (lMCI). Later, in ADNI2, a subjective memory complaint (SMC) group was added (Beckett et al., 2015). Similarly to ADNI1, both AIBL and CADDementia sets were composed of CN, MCI, and AD stages, whereas both MIRIAD and OASIS sets contained only CN and AD.

**Table 2:** Datasets considered in this study.

**Dataset**	**Number of MRI images**	**References**
ADNI	18,303	Mueller et al., 2005;
(ADNI1, ADNIGO, ADNI2)		Beckett et al., 2015
AIBL	1,098	Ellis et al., 2009
CADDementia	384	Bron et al., 2015
MIRIAD	708	Malone et al., 2013
OASIS	3,056	Marcus et al., 2007, 2009

Since one of our main goals for this research was achieving a good result in the CADDementia challenge, we adopted only equivalent diagnoses. As such, eMCI and lMCI stages were grouped along with MCI, and SMC was not considered. From these datasets, we downloaded all available raw *T*_1_-weighted sMRI scans associated with Alzheimer's, i.e., we did not consider any pre- or post-processed image.

To isolate possible confounding factors, we made a distinction between MP-RAGE and IR-SPGR/IR-FSPGR sequences (Lin et al., 2006; Jack et al., 2008), and aggregated different data sources and sequence techniques in steps. While all ADNI sets had both MP-RAGE and IR-SPGR/IR-FSPGR, AIBL and OASIS had only MP-RAGE, and MIRIAD had only IR-FSPGR. The resulting datasets are described in Table 3, and detailed in Table 4.

**Table 3:** Datasets assembled in this study.

**Resulting dataset**	**Overarching sets**	**MP-RAGE only**	**Final number of MRI images**
Dataset 1	ADNI1	Yes	9,149
Dataset 2	All ADNI	Yes	15,885
Dataset 3	All ADNI	No	18,303
Dataset 4	All	No	23,165

**Table 4:** Datasets summaries: number of subjects, number of images, descriptive age statistics, image-wise percentage of females (vs. males) and image-wise percentage of 1.5 T field strength (vs. 3.0 T).

**Dataset**	**Subjs**.	**Group**	**Images**	**Age (years)**				**Female (%)**	**1.5 T(%)**
				**Med**	**Avg ± Std**	**Min**	**Max**		
Dset. 1	845	All	9,149	76.6	76.3 ± 6.9	54.6	93.0	42.2	82.2
		CN	2,701	76.7	77.2 ± 5.1	60.0	92.8	50.2	80.5
		MCI	4,845	76.5	76.0 ± 7.4	54.6	90.9	35.3	83.0
		AD	1,603	76.5	76.1 ± 7.9	55.2	93.0	49.5	82.5
Dset. 1 Train.	591	All	6,314	76.5	76.2 ± 6.9	54.6	93.0	43.4	82.6
		CN	1,809	77.2	77.3 ± 4.9	60.0	90.8	49.5	81.3
		MCI	3,399	76.1	75.7 ± 7.3	54.6	90.9	36.3	83.0
		AD	1,106	75.9	76.1 ± 7.9	55.2	93.0	55.3	83.5
Dset. 1 Val.	84	All	951	76.4	75.8 ± 6.8	56.2	89.2	40.5	82.8
		CN	301	75.7	76.5 ± 4.8	65.2	88.6	58.5	79.7
		MCI	501	78.2	76.7 ± 6.7	56.2	89.2	28.5	83.8
		AD	149	72.0	71.2 ± 8.6	56.5	85.0	44.3	85.2
Dset. 1 Test	170	All	1,884	77.2	77.0 ± 6.9	56.7	92.8	38.7	80.4
		CN	591	76.2	77.2 ± 5.6	63.3	92.8	47.9	78.5
		MCI	945	77.7	76.5 ± 7.8	56.7	90.9	35.1	82.4
		AD	348	79.7	78.0 ± 6.3	63.1	87.7	33.0	78.2
Dset. 2	1503	All	15,885	75.8	75.4 ± 7.3	54.6	95.8	44.0	53.3
		CN	4,646	76.8	76.9 ± 5.8	56.3	95.8	50.0	56.5
		MCI	8,940	75.0	74.6 ± 7.7	54.6	93.5	40.0	50.5
		AD	2,299	76.4	75.8 ± 7.8	55.2	93.0	47.5	57.5
Dset. 3	1715	All	18,303	75.8	75.5 ± 7.4	54.6	95.8	43.5	48.2
		CN	5,361	76.7	76.9 ± 6.0	56.3	95.8	50.0	52.5
		MCI	10,306	75.0	74.6 ± 7.7	54.6	93.6	39.5	45.5
		AD	2,636	76.2	75.8 ± 7.9	55.2	93.0	45.9	50.2
Dset. 4	2984	All	23,165	75.0	73.5 ± 11.7	18.0	98.0	46.5	55.5
		CN	8,462	75.0	71.3 ± 16.1	18.0	97.0	53.9	62.8
		MCI	10,460	75.0	74.7 ± 7.7	54.6	96.0	39.6	45.1
		AD	4,243	75.4	75.3 ± 7.9	55.0	98.0	48.4	66.3
CADD. Train.	30	All	30	65.0	65.2 ± 6.9	54.0	80.0	43.3	0.0
		CN	12	62.0	62.3 ± 6.1	55.0	79.0	25.0	0.0
		MCI	9	68.0	68.0 ± 8.2	54.0	80.0	44.4	0.0
		AD	9	67.0	66.1 ± 5.0	57.0	75.0	66.7	0.0
CADD. Test	354	All	354	65.0	65.1 ± 7.8	46.0	88.0	39.8	0.0

*AD, Alzheimer's disease; Avg, average; CADD., CADDementia; CN, cognitively normal; Dset., dataset; Max, maximum; Med, median; MCI, mild cognitive impairment; Min, minimum; Std, standard deviation; Subjs., subjects; Train., training; Val., validation*.

For each dataset, we created training, validation, and test splits. In Dataset 1, we randomly split the corresponding subjects, trying to keep the original age, sex, and diagnostic stratification across each set, with 70% of subjects for training, 10% for validation, and 20% for testing. In each subsequent dataset, we first assigned images from previous subjects to the respective set, then we proceeded with the stratified random split considering only new subjects.

### 3.2. Metrics and Optimization

The primary evaluation measure we considered herein was classification accuracy, which is the number of correctly classified samples divided by the number of all samples. Even though this performance value does not take into account class priors, the challenge organization deemed class sizes insignificantly different, therefore regarding this metric as a better approach for overall classification accuracy. Additionally, the receiver operating characteristic (ROC) curve and the respective area under the curve (AUC) were also considered, as they provide metrics that are independent of the threshold chosen for classification. Also, since AUC does not traditionally depend on class sizes, we adopted an AUC measure that does not rely on class priors. Finally, the true positive fraction (TPF), the number of correctly classified samples of a given class divided by the number of all samples from that class, was calculated for each class. According to the authors, TPFs for diseases (AD and MCI) can be interpreted as the two-class sensitivity, while TPF for CN corresponds to the two-class specificity.

As we optimized and trained our networks, we compared them and selected the best ones using the average of TPFs, since it more closely relates to the accuracy and does not depend on class priors. To perform the training process, we used Adam optimizer (Kingma and Ba, 2014) with default parameters (i.e., β_1_ = 0.9, β_2_ = 0.999, and ϵ = 10^−8^). With a small sample of images, we empirically decided to begin with a learning rate of α = 10^−4^, and settled to a batch size of three (for VGG architectures) or nine (for all the others), mainly due to GPU memory limitations, even though we only used GPUs with 12 GB of dedicated memory. Finally, we adopted Glorot uniform initialization (Glorot and Bengio, 2010) with scaling factor of $\sqrt{2}$.

## 4. Results and Discussions

Fairness, accountability, and transparency (FAT) have become increasingly essential aspects of machine learning (Goodman and Flaxman, 2016). For example, laudable efforts include explaining algorithmic decisions, making an effort to understand sources of error and uncertainty, and creating auditable systems (Diakopoulos et al., 2017).

Given the expectations described above, we will now discuss further details of our study. We better describe our optimization process, specifying the steps to handle overfitting problems. Then, we report performance results, including previously described metrics, along with efficiency measurements. Finally, we discuss our best CNN model, providing further insights into its functionality, and how it processes data to make predictions.

### 4.1. Optimization

As stated earlier, we determined the initial learning rate of α = 10^−4^, and varied some configurations in each architecture to achieve the best accuracy in the CADDementia training set. These options included regularization with L1 and L2 norms, regularization strength λ, number of units in fully-connected layers, dropout rates, batch size, and multi-class hinge loss (instead of the traditional categorical cross-entropy loss).

The parameters for regularization strength, number of units, and dropout rates were also used for regularization, acting as trade-offs between model complexity and bias, thus managing the probability to overfit. Overfitting was a significant concern for us due to the large size of our networks and a relatively small amount of data. The different batch size was an experiment to compare the behavior of all networks with the same batch size of three. Given that support vector machine (SVM; Cortes and Vapnik, 1995) classifiers usually present reasonable results, and have successfully been used to identify Alzheimer's biomarker previously (Magnin et al., 2009), we also experimented with the multi-class hinge loss.

In general, we varied regularization strength λ in powers of 10, between 10^−5^ and 10^2^, number of units in fully-connected layers in powers of 2, between 32 and 2, 048, and dropout rate with steps of 10 percentage points, between 40 and 90%, including 95, 99, and 99.9%. Note that some networks had specific parameters, i.e., these variations did not apply to all evaluated architectures. We followed a greedy approach, by first tuning regularization strength with L2 norm, followed by several units, and then dropout rates. Next, we evaluated batches of size three for all networks, L1 norm, multi-class hinge loss, and, finally, larger datasets.

To form a balanced batch, the same number of samples was consistently selected from each class. In each epoch, we randomly sampled each class, limited by the class with fewer images. We worked with a total batch size of either three or nine samples, depending on the network architecture.

We observed that, at some point, most networks underfit or overfit, presenting erratic metrics, with high variations between epochs. To overcome this issue, we applied the early stopping to interrupt the optimization before the model began to overfit. After 50 epochs without further improvement in average TPF over the validation set, the training was stopped. The model was optimized for up to 200 epochs.

### 4.2. Performance

We first analyzed the efficiency of our processing pipeline, divided into brain image, CNN, and domain adaptation stages.

**Figure 2:**
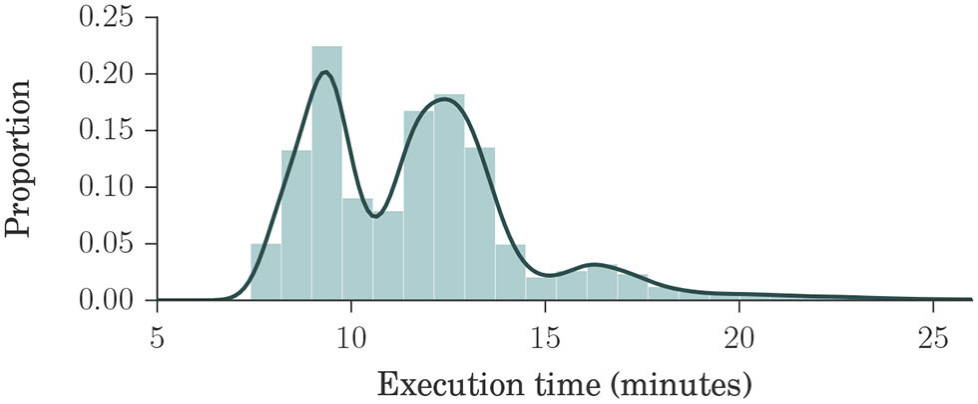
Histogram and kernel density estimation plots of brain extraction and normalization times for Dataset 4, in minutes. Dataset 4 is our largest, composed of ADNI1, ADNIGO, ADNI2, AIBL, MIRIAD, and OASIS datasets, with a total of 23,165 volumes. These plots show that the processing times ranged mostly between 7 and 15 min, with an average of about 12 min, indicating that our method is fast.

Figure 2 shows a histogram and a kernel density estimation of the execution time of brain extraction and normalization steps for Dataset 4, which is our largest, containing 23,165 volumes. Interestingly, only 151 images (0.7%) took longer than 25 min to process. Each process used two cores in a shared cluster of commodity hardware, such as Intel® Xeon® CPU E5645 at 2.40 GHz, and around 2 GB of RAM.

To train our CNNs, we used three different models of NVIDIA GPUs: GeForce GTX TITAN X (Maxwell microarchitecture), Tesla K40c, and Tesla K80. Training usually lasted for about 100 epochs, taking around 4 days to complete. We performed a total of 121 experiments. Inference time for our best network (VGG 512) was <1 s. The grid search for domain adaptation was completed in under one minute, and the classification of all 354 samples from CADDementia test set was accomplished in about 1 ms.

In summary, our method is expected to provide a response in <15 min, with extreme cases taking a little longer than 2 h. This processing time contrasts with the current best method in CADDementia challenge, which requires 19 h of computation (Sørensen et al., 2017). In other words, our method is nearly 10× faster, considering the worst-case scenario, or almost 80× faster, on average.

Regarding performance metrics in terms of results, we present our best configuration for each network architecture in Table 5. The best VGG had 512 units in each fully-connected layer, and the best ResNet used the projection shortcut (B). We also included our main optimization metric—average TPF (avgTPF)—for the training set of CADDementia, in which the top value was 75.9%, translating to 76.7% in accuracy. All of these results were found while optimizing the networks with Dataset 1.

**Table 5:** Performance results (average true positive fraction, labeled avgTPF) of our best CNN architectures and respective configurations found in optimization experiments.

**Architecture**	**avgTPF (%)**	**Norm**	**λ**	**Dropout (%)**
LeNet-5	56.5	L2	10^−2^	40
VGG 512	75.9	L2	10^−4^	50
GoogLeNet	58.3	L1	10^−3^	80
ResNet B	60.2	L2	10^−2^	−

As initially expected, L2 norm provided the best results for almost all architectures. The best GoogLeNet using L2 achieved 57.4% average TPF, close to the one using L1 (58.3%), while the L1 norm performed considerably worse for the other networks. ResNet with identity shortcuts (A) achieved 57.4%, which is slightly inferior to the projection shortcut (B), with 60.2%, a similar difference found in the original work (He et al., 2016). We hypothesize that deeper architectures did not achieve the highest scores because they tend to do better in more massive datasets, which we did not have.

A batch size of three (instead of nine) only produced significantly worse results, indicating that our best VGG model could potentially achieve even better results if we used GPUs with larger memory or a multi-GPU framework implementation. Similarly, multi-class hinge loss did not improve our results. Most surprisingly, Dataset 1, our smallest, presented the best performances, and Dataset 2 achieved an average TPF as high as 72.2%. We hypothesize that this happened due to the higher diversity of data sources and conditions in more massive sets, indicating that a smaller but more cohesive dataset should be sufficient for optimization.

VGG 512 was our best network model, and the respective performance metrics are shown in Table 6. We named our CNN approach ADNet (Alzheimer's Disease Network), with the domain adaptation method ADNet-DA, and submitted our prediction scores to the CADDementia challenge. Currently, there are 48 different submissions, ours included^3^. Similar to previous results (Esmaeilzadeh et al., 2018; Abrol et al., 2020), we also observed some overfitting in the training data. However, the performance differences in Dataset 1 between validation and test sets were small, indicating that we appropriately mitigated this problem.

**Table 6:** Multiple performance results of our best CNN, in percentage.

**Model**	**Dataset**	**Split**	**Accuracy**	**TPF**			**AUC**			
				**CN**	**MCI**	**AD**	**All**	**CN**	**MCI**	**AD**
ADNet	Dataset 1	Train.	60.6	89.6	36.7	86.8	87.9	90.3	80.6	88.8
		Val.	44.1	71.1	22.4	62.4	68.9	72.2	56.9	72.5
		Test	43.6	67.3	21.1	64.7	68.0	73.9	57.0	68.9
ADNet	CADD	Train.	76.7	83.3	55.6	88.9	90.3	92.1	83.1	96.3
		Test	51.4	77.5	27.9	46.6	68.5	70.5	61.2	73.6
AD-DNAet	CADD	Train.*	76.7	75.0	55.6	100.0	88.5	90.7	79.4	95.8
		Train.	90.0	83.3	88.9	100.0	98.0	95.8	97.9	100.0
		Test	52.3	68.2	37.7	49.5	70.9	72.8	60.5	79.0

AD, Alzheimer's disease; AUC, area under the receiver operating characteristic curve; CN, cognitively normal; CADD, CADDementia; CNN, convolutional neural network; MCI, mild cognitive impairment; Train., training; TPF, true-positive fraction; Val., validation; Train.*, leave-one-out cross-validation results.

In general, ADNet presented promising results in the CADDementia training set. The low TPF in the MCI group was expected, since classifying MCI subjects solely on their sMRI represents a challenge (Albert et al., 2011), also observed in a number results of CADDementia submissions (Bron et al., 2015). However, the decrease in MCI and AD TPFs between training and test sets was higher than expected. As such, this method achieved an interesting two-class specificity, with a modest two-class sensitivity, meaning it is better suited for determining healthy patients. Regarding accuracy in the test set, ADNet ranked 25th, tied with two other systems, outperforming 22 submissions. Besides, this result was only statistically different, with a 95% confidence interval from the first and the last three systems. Considering that we were the first group that did not use any domain-specific information for this task, we can claim that our CNN method was able to learn meaningful patterns automatically.

As for the domain adaptation approach, we extracted 512 features from the second-to-last layer of ADNet, and then we performed a grid search on the parameters of a logistic regression classifier. Using the best parameters found (most importantly, *C* = 0.001), we optimized this classifier on the complete training set and applied it to output classification probabilities for each sample from the challenge. We also submitted these predictions to CADDementia, naming it ADNet-DA (ADNet with domain adaption). The corresponding results are also indicated in Table 6. This method ranked 21st, outperforming 27 submissions, with a statistical difference from the first and the last four systems.

**Figure 3:**
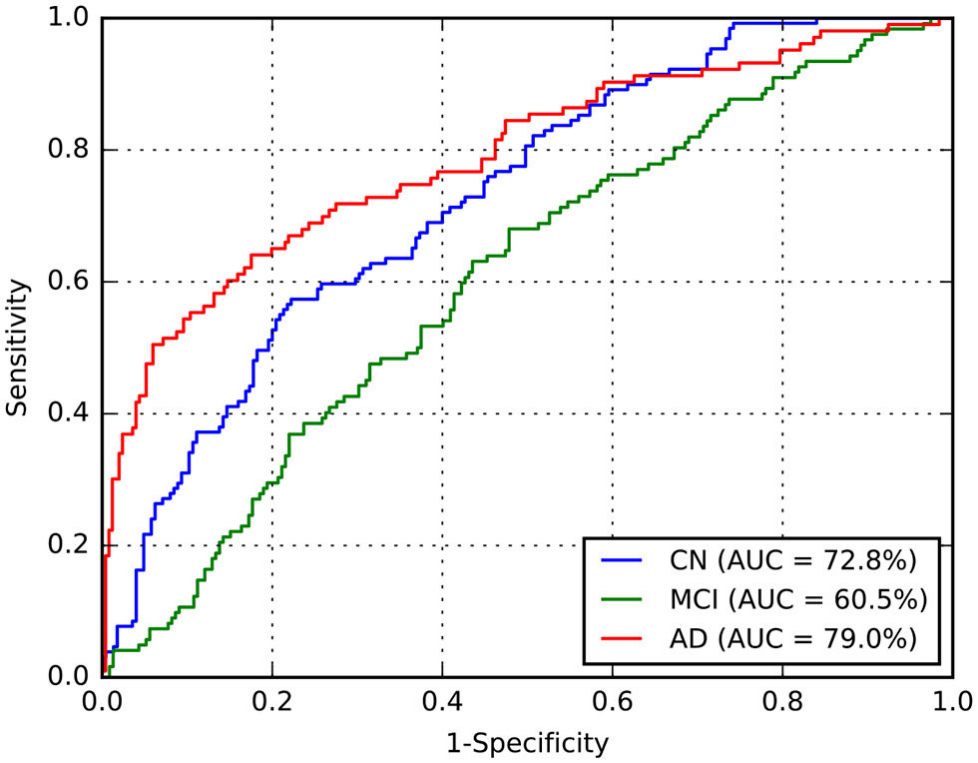
Receiver operating characteristic (ROC) curve for ADNet-DA, provided by CADDementia. AD, Alzheimer's disease; AUC, area under the receiver operating characteristic curve; CN, cognitively normal; MCI, mild cognitive impairment.

Considering this approach, we reported the leave-one-out cross-validation results in the training set while performing a grid search, and also the results in this same set after the last optimization with all training samples. As expected, developing and evaluating a system on the same data overestimated its generalization performance; however, even our cross-validation attempt did not significantly improve our estimations for the test set. In comparison with ADNet, ADNet-DA improved both MCI and AD TPFs, while decreasing CN TPF, with an overall improvement of almost one percentage point in accuracy. These results indicate that domain adaptation was indeed an important technique. The corresponding ROC for CADDementia test set is displayed in Figure 3, and the respective confusion matrix is in Table 7.

**Table 7:** Confusion matrix (in percentage) for ADNet-DA, provided by CADDementia.

		**Prediction**		
		**CN**	**MCI**	**AD**
**Actual**	**CN**	68.2	25.6	6.2
	**MCI**	51.6	37.7	10.7
	**AD**	29.1	21.4	49.5

*Values are adjusted relative to the actual class, i.e., divided by the row sum. This way, the main diagonal represents the true positive fraction (TPF) for each class. AD, Alzheimer's disease; CN, cognitively normal; MCI, mild cognitive impairment*.

Though Dolph et al. (2017) pioneered deep learning on this challenge, we are the first (to our knowledge) to propose an end-to-end training deep 3D CNN for the multiclass AD biomarker identification task in CADDementia. One of their systems ranked 7th, with 56.8% accuracy, while the other ranked 25th, tied with ADNet on 51.4%. Our ADNet-DA method was able to outperform a deep-learning system that uses domain-specific information, which demonstrates the effectiveness of the approach proposed in this work.

### 4.3. Accountability

Understanding the decision-making process of a machine-learning algorithm has become crucial lately, especially in medicine. For practical application, an algorithm must present good performance results and also demonstrate how predictions are generated. The explicability requirement has become even more critical in recent years with rules such as the General Data Protection Regulation (GDPR), which also brought explainable artificial intelligence (XAI) to the spotlight (Goodman and Flaxman, 2016).

Explaining what and how a neural network has learned is an open problem, with a rapidly evolving research field. In order to better understand what our model is analyzing in brain images and how it is done, we experimented with a number of visualization approaches, considering the most used techniques in accountable machine learning for neural networks. Some of these approaches were also recently explored by Rieke et al. (2018).

Similarly to Krizhevsky et al. (2012), we analyzed the filters from our first convolutional layer. While their kernels were of size 11 × 11 × 3, presenting some interesting smooth and colorful patterns, our kernels are 3 × 3 × 3 in grayscale, producing less than ideal images for visualization.

Another traditional approach for visualization is to show outputs of activation functions from the network, after processing an input. Activation is simply the result of a mathematical function. These outputs represent some of the initial patterns that the network learned to be the most relevant for this task. These outputs are then non-linearly combined with additional and more complex patterns before the final classification.

Occlusion is a technique to visualize how and where the input image affects the result of the network. The basic idea is to systematically hide (occlude) some regions of the input image, preventing the network from becoming activated in these specific regions, and then storing the probabilities output. Given a class of interest, for instance AD, it is possible to create a heatmap with the corresponding prediction for each occluded region, where most important regions will present highest impact (with low probability), due to the occlusion. This technique was initially proposed by Zeiler and Fergus (2014).

There are different ways to hide a region of the input image and avoid activations in a network. The most straightforward and most direct would be to set input values to their respective averages, which, in our case, is zero. Considering images in a range from zero to 255, it is possible to occlude with the average value (gray), with zero (black), with 255 (white), and even more sophisticated approaches, such as different forms of noise.

Finally, we investigated an approach that more closely related to the actual output decision of the network. For that, we calculated the gradient of the network concerning the input, which is used to update the network's internal parameters. These gradients may also be plotted and interpreted as how much the output is affected by changes in input values; however, this simple approach produces rather noisy visualizations. An improvement to this technique, called deconvolution, was proposed (Zeiler and Fergus, 2014) and can be interpreted as reversing the operations performed by the network. Even though this is an interesting approach, the guided backpropagation method (Springenberg et al., 2014) produces even sharper visualizations. Interestingly, guided backpropagation combines calculations from both backpropagation and deconvolution, resulting in more detailed images.

**Figure 4:**
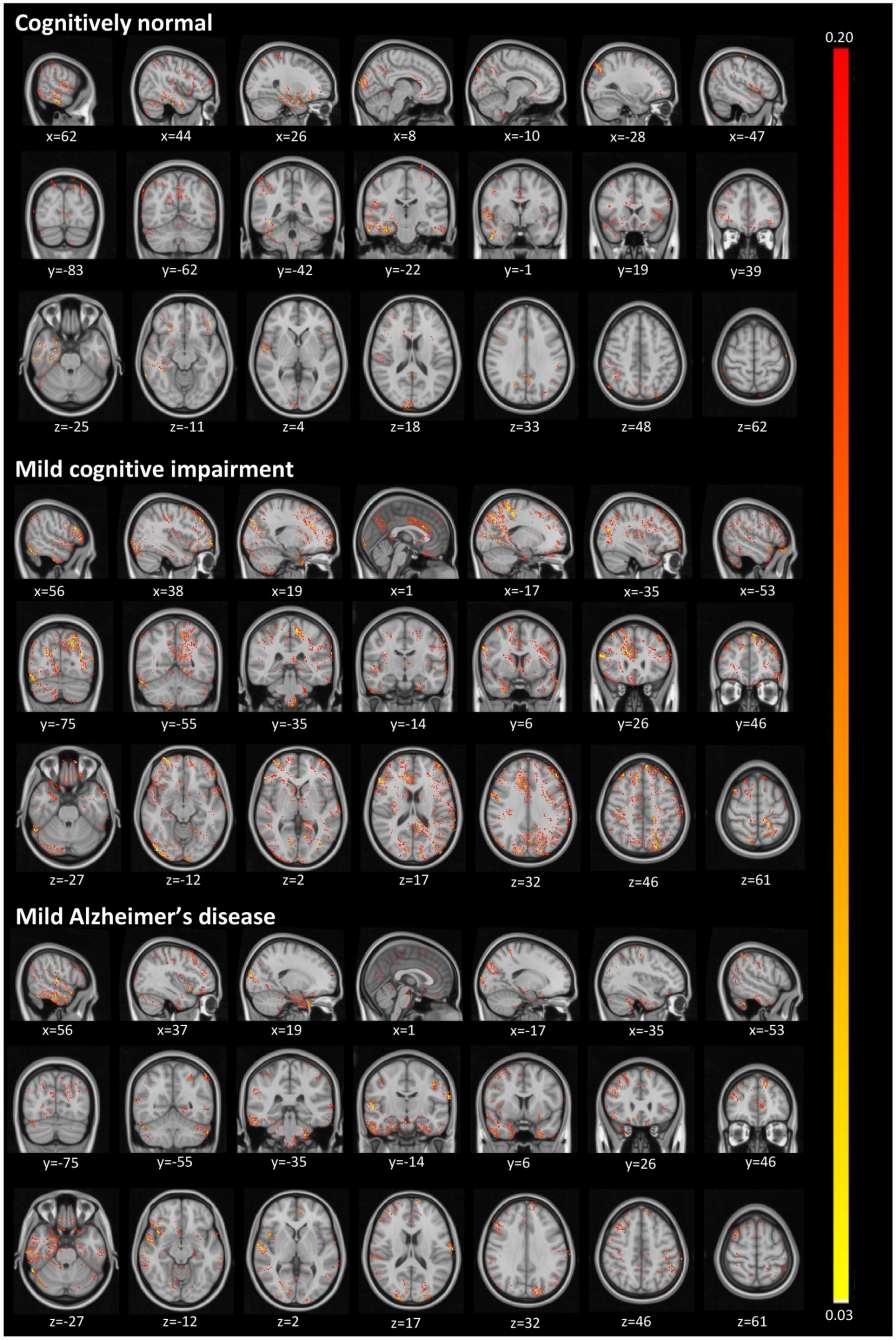
Activated regions from the guided backpropagation method for each group. Activations are displayed in *hot* colormap overlaid onto the MNI template. *Hotter* regions mean a more significant effect on the prediction output. Colors only represent the relative importance of each voxel, having no direct meaning associated to their absolute values.

Figure 4 illustrates activated areas for each group, where brighter regions mean a larger effect on the prediction output. For the CN group, we can see activations distributed in a diffuse pattern, but mainly restricted to the cortex in the right temporal lobe (predominantly in the medial temporal gyrus and the parahippocampal gyrus), the central portion of the occipital lobe, the posterior cingulum, and the posterior parietal cortex. For MCI, activations occurred mainly in the left posterior parietal cortex, the right anterior cingulum, and the right dorsolateral prefrontal cortex. For AD, more significant activations were detected in the left posterior parietal cortex, right temporal pole, cerebellum, and more diffusively in the spherical surface of the brain.

The diffuse pattern of activations in all groups (mainly in temporal and posterior regions of the brain) can be interpreted in the context of neuroimaging findings in the field of AD. Although no single structure can differentiate AD patients from CN subjects, atrophy in temporal regions seems to be an inevitable process in the disease. The medial temporal lobe regions might be the first affected in the course of the disease, presenting very early signs of neurodegeneration (Karas et al., 2004), which correlate with clinical symptoms even in the prodromal stage, i.e., MCI (Frisoni et al., 2010). As in pathophysiological aspects, the temporal regions mainly present intracellular aggregates of hyperphosphorylated tau protein, which are associated with reduced gray matter density (Thomann et al., 2009). Another signature of AD, extracellular amyloid β-protein (Aβ) deposition in the form of plaques, is mainly observed in the midline regions (posterior cingulate and medial prefrontal cortices), and parietal areas. Longitudinal studies have shown that these areas not only atrophy at the mild stage of AD (Weiler et al., 2015), but they continue to degenerate at a rate of about 2–4% per year (Thompson et al., 2003; Leow et al., 2009). Thus, we were not surprised to find larger activations in those regions classically affected by the disease.

For our last visualization technique, our motivation was to understand how our data samples were spatially distributed within internal feature representations of our network, in order to determine whether these representations were really helpful to discriminate between each class. To plot our data from this high-dimensional space, we first projected them into two dimensions using the t-distributed stochastic neighbor embedding (t-SNE; Maaten and Hinton, 2008), with principal component analysis (PCA) initialization. Considering the outputs from a specific layer of our network, we generated an embedding with all training and test data in CADDementia, and then colored training samples according to each respective class. It is important to remark that this projection did not use label information from training data, which was used solely to color our plots.

**Figure 5:**
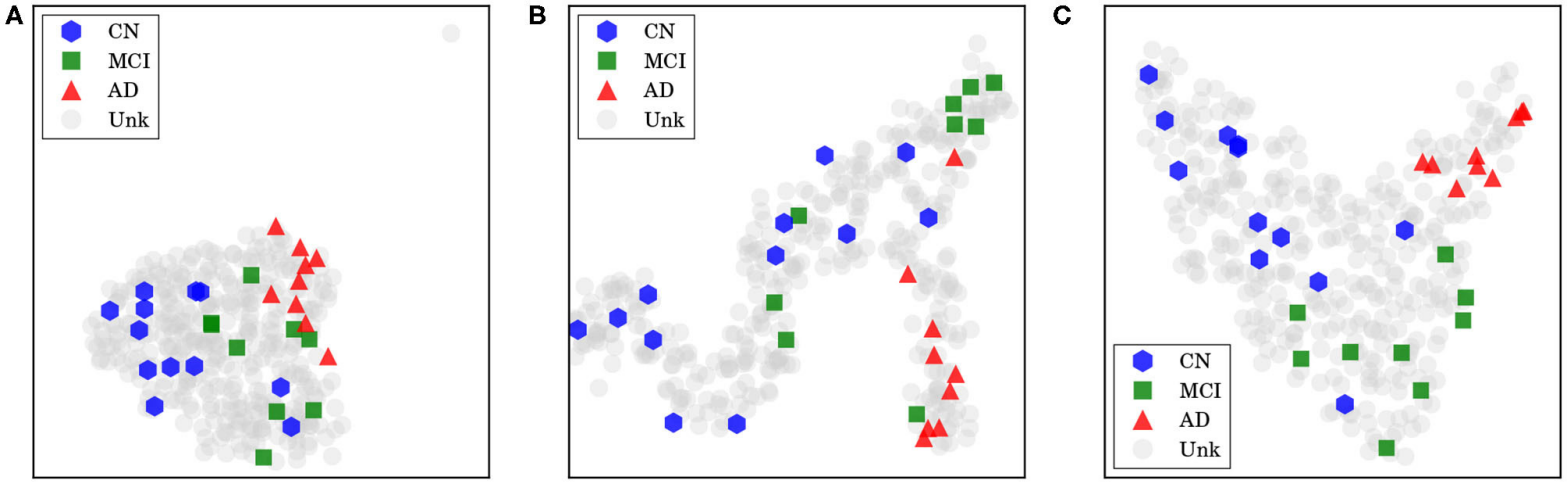
Features and probabilities visualizations with 2D t-SNE projections. AD, Alzheimer's disease; CN, cognitively normal; MCI, mild cognitive impairment; Unk, unknown. **(A)** Visualization with 2D t-SNE projections using 512-dimensional features extracted from the second-to-last layer. **(B)** Visualization with 2D t-SNE projections using the 3-dimensional ADNet probabilities. **(C)** Visualization with 2D t-SNE projections using the 3-dimensional ADNet-DA probabilities.

First, we extracted features from the second-to-last layer of our network, traditionally used to transfer learning and domain adaptation, with 512 dimensions. Then, we considered the final layer from ADNet that outputs classification probabilities, with 3 dimensions, and the probability outputs from ADNet-DA. Resulting embeddings are present in Figure 5. Considering ADNet, even though t-SNE (Maaten and Hinton, 2008) did not use any label information, training data points were better grouped in an internal feature representation space rather than in the probability output space, indicating that the softmax classifier used in the network did not perform as well as it could. From these plots, we can also see that probabilities from ADNet-DA are better distributed in comparison with ADNet, especially for the AD group, while there was a smaller confusion for MCI.

## 5. Conclusions

Using data from ADNI, we optimized a 3D CNN with the whole brain image as input and achieved the best accuracy with a network architecture based on VGG (Simonyan and Zisserman, 2014). Our method, named ADNet, outperformed several other systems in the prior art. Additionally, our method with domain adaptation, called ADNet-DA, reached 52.3% in accuracy on the CADDementia challenge test set, outperforming most of the submissions to this challenge. Our approach is completely automatic (i.e., does not require additional information input and manual intervention), and is considerably fast (around 80 times faster than CADDementia winning methods).

Importantly, whereas all other submissions used prior information from the disease (e.g., hippocampal volume, demographic information), our method did not use any domain-specific knowledge from AD. For that reason, we believe it could be applied to other disorders that could benefit from the CAD system using sMRI as input data. We understand that our approach can be used to find meaningful patterns within data, corroborate previous findings by specialists, assist in diagnosis scenarios, and eventually help identify patterns for diseases other than AD. Our conclusions are supported by our explainable artificial intelligence (XAI) techniques, including accountability visualizations.

Future work could investigate XAI techniques to understand brain regions involved in the decision-making process, and cross-match highlighted regions with specialists knowledge, to see how one can complement the other in refining the technique. Finally, it would be interesting to incorporate patients' history data to enrich the information present in MRIs, to drive the decision process and to tie it with patients' backgrounds.

## Data Availability Statement

Generated datasets are a compilation of public data, which require specific data use agreements. Full information on accessing this data can be found in the acknowledgments of this article.

## Ethics Statement

Ethical review and approval was not required for the study on human participants in accordance with the local legislation and institutional requirements. Written informed consent for participation was not required for this study in accordance with the national legislation and the institutional requirements.

## Author Contributions

AR and MW coordinated this research and helped in defining our method and experimental design. RC assisted with the neurological area and with the interpretation of our explainable AI visualizations. GF developed our method and dealing directly with the data and the experiments. RP contributed to the manuscript writing. All authors contributed to the article and approved the submitted version.

## Conflict of Interest

The authors declare that the research was conducted in the absence of any commercial or financial relationships that could be construed as a potential conflict of interest.

## Footnotes

^1^https://github.com/gfolego/alzheimers

^2^Specifically, scripts *antsBrainExtraction.sh* and *antsRegistrationSyNQuick.sh* of version 2.1.0.

^3^https://caddementia.grand-challenge.org/results_all/ [Online; accessed 2020-05-14].

## References

[B1] Abrol A. Bhattarai M. Fedorov A. Du Y. Plis S. Calhoun V. (2020). Deep residual learning for neuroimaging: an application to predict progression to Alzheimer's disease. J. Neurosci. Methods 339:108701. 10.1016/j.jneumeth.2020.108701 32275915 PMC7297044

[B2] Albert M. S. DeKosky S. T. Dickson D. Dubois B. Feldman H. H. Fox N. C.. (2011). The diagnosis of mild cognitive impairment due to Alzheimer's disease: recommendations from the national institute on aging-Alzheimer's association workgroups on diagnostic guidelines for Alzheimer's disease. Alzheimer's Dement. 7, 270–279. 10.1016/j.jalz.2011.03.008 21514249 PMC3312027

[B3] Al-Rfou R. Alain G. Almahairi A. Angermueller C. Bahdanau D. Ballas N. (2016). Theano: a Python framework for fast computation of mathematical expressions. ArXiv e-prints. arXiv:1605.02688.

[B4] Avants B. B. Tustison N. Song G. (2009). Advanced normalization tools (ANTs). Insight J. 2, 1–35. Available online at: http://hdl.handle.net/10380/3113

[B5] Avants B. B. Tustison N. J. Song G. Cook P. A. Klein A. Gee J. C. (2011). A reproducible evaluation of ANTs similarity metric performance in brain image registration. NeuroImage 54, 2033–2044. 10.1016/j.neuroimage.2010.09.025 20851191 PMC3065962

[B6] Beckett L. A. Donohue M. C. Wang C. Aisen P. Harvey D. J. Saito N. (2015). The Alzheimer's disease neuroimaging initiative phase 2: increasing the length, breadth, and depth of our understanding. Alzheimer's Dement. 11, 823–831. 10.1016/j.jalz.2015.05.004 26194315 PMC4510463

[B7] Bron E. E. Smits M. van der Flier W. M. Vrenken H. Barkhof F. Scheltens P.. (2015). Standardized evaluation of algorithms for computer-aided diagnosis of dementia based on structural mri: the CADDementia challenge. NeuroImage 111, 562–579. 10.1016/j.neuroimage.2015.01.048 25652394 PMC4943029

[B8] Collins D. L. Zijdenbos A. P. Baaré W. F. Evans A. C. (1999). Animal+insect: improved cortical structure segmentation, in Information Processing in Medical Imaging 16th International Conference, IPMI'99, eds Kuba A. Šáamal M. Todd-Pokropek A. (Visegrád: Springer), 210–223. 10.1007/3-540-48714-X_16

[B9] Cortes C. Vapnik V. (1995). Support-vector networks. Mach. Learn. 20, 273–297. 10.1007/BF00994018

[B10] Diakopoulos N. Friedler S. Arenas M. Barocas S. Hay M. Howe B. (2017). Principles for Accountable Algorithms and a Social Impact Statement for Algorithms. FAT/ML. (Accessed May 14, 2020).

[B11] Dieleman S. Schlüter J. Raffel C. Olson E. Sønderby S. K. Nouri D. (2015). Lasagne: First Release. Geneva: Zenodo 10.5281/zenodo.27878

[B12] Dolph C. V. Alam M. Shboul Z. Samad M. D. Iftekharuddin K. M. (2017). Deep learning of texture and structural features for multiclass Alzheimer's disease classification, in International Joint Conference on Neural Networks (IJCNN) (Anchorage, AK), 2259–2266. 10.1109/IJCNN.2017.7966129

[B13] Ellis K. A. Bush A. I. Darby D. De Fazio D. Foster J. Hudson P.. (2009). The Australian imaging, biomarkers and lifestyle (AIBL) study of aging: methodology and baseline characteristics of 1112 individuals recruited for a longitudinal study of Alzheimer's disease. Int. Psychogeriatr. 21, 672–687. 10.1017/S1041610209009405 19470201

[B14] Esmaeilzadeh S. Belivanis D. I. Pohl K. M. Adeli E. (2018). End-to-end Alzheimer's disease diagnosis and biomarker identification, in Machine Learning in Medical Imaging, eds Shi Y. Suk H.-I. Liu M. (Cham: Springer International Publishing), 337–345. 10.1007/978-3-030-00919-9_39 PMC7440044 32832936

[B15] Evans D. A. Funkenstein H. H. Albert M. S. Scherr P. A. Cook N. R. Chown M. J.. (1989). Prevalence of Alzheimer's disease in a community population of older persons: higher than previously reported. JAMA 262, 2551–2556. 10.1001/jama.1989.03430180093036 2810583

[B16] Folego G. (2018). Adnet: computer-aided diagnosis for Alzheimer's disease using whole-brain 3D convolutional neural network (Master's thesis). University of Campinas, Campinas, Brazil.

[B17] Fonov V. Evans A. C. Botteron K. Almli C. R. McKinstry R. C. Collins D. L. (2011). Unbiased average age-appropriate atlases for pediatric studies. NeuroImage 54, 313–327. 10.1016/j.neuroimage.2010.07.033 20656036 PMC2962759

[B18] Fonov V. S. Evans A. C. McKinstry R. C. Almli C. Collins D. (2009). Unbiased nonlinear average age-appropriate brain templates from birth to adulthood. NeuroImage 47(Suppl. 1):S102 10.1016/S1053-8119(09)70884-5

[B19] Frisoni G. B. Fox N. C. Jack C. R. Scheltens P. Thompson P. M. (2010). The clinical use of structural MRI in Alzheimer disease. Nat. Rev. Neurol. 6, 67–77. 10.1038/nrneurol.2009.215 20139996 PMC2938772

[B20] Glorot X. Bengio Y. (2010). Understanding the difficulty of training deep feedforward neural networks, in 13th International Conference on Artificial Intelligence and Statistics (AISTATS), eds Teh Y. W. Titterington M. (Sardinia: PMLR), 249–256

[B21] Goodman B. Flaxman S. (2016). European Union regulations on algorithmic decision-making and a “right to explanation”. ArXiv e-prints. 10.1609/aimag.v38i3.2741

[B22] He K. Zhang X. Ren S. Sun J. (2016). Deep residual learning for image recognition, in 2016 IEEE Conference on Computer Vision and Pattern Recognition (CVPR) (Las Vegas, NV), 770–778. 10.1109/CVPR.2016.90

[B23] Hosseini-Asl E. Ghazal M. Mahmoud A. Aslantas A. Shalaby A. M. Casanova M. F.. (2018). Alzheimer's disease diagnostics by a 3D deeply supervised adaptable convolutional network. Front. Biosci. 23, 584–596. 10.2741/4606 28930562

[B24] Ioffe S. Szegedy C. (2015). Batch normalization: accelerating deep network training by reducing internal covariate shift, in The 32nd International Conference on Machine Learning (ICML 2015), eds Blei D. Bach F. (Lille), 448–456.

[B25] Jack C. R. Jr. Bernstein M. A. Fox N. C. Thompson P. Alexander G. Harvey D.. (2008). The Alzheimer's disease neuroimaging initiative (ADNI): MRI methods. J. Magn. Reson. Imaging 27, 685–691. 10.1002/jmri.21049 18302232 PMC2544629

[B26] Karas G. Scheltens P. Rombouts S. Visser P. van Schijndel R. Fox N.. (2004). Global and local gray matter loss in mild cognitive impairment and Alzheimer's disease. NeuroImage 23, 708–716. 10.1016/j.neuroimage.2004.07.006 15488420

[B27] Kingma D. P. Ba J. (2014). Adam: A method for stochastic optimization. arXiv. arXiv:1412.6980.

[B28] Korolev S. Safiullin A. Belyaev M. Dodonova Y. (2017). Residual and plain convolutional neural networks for 3D brain MRI classification, in IEEE International Symposium on Biomedical Imaging (Melbourne, VIC), 835–838. 10.1109/ISBI.2017.7950647

[B29] Krizhevsky A. Sutskever I. Hinton G. E. (2012). Imagenet classification with deep convolutional neural networks, in Advances in Neural Information Processing Systems 25, eds Pereira F. Burges C. J. Bottou L. Weinberger K. Q. (Stateline; Lake Tahoe, NV: Curran Associates, Inc.), 1097–1105.

[B30] LeCun Y. Boser B. Denker J. S. Henderson D. Howard R. E. Hubbard W. (1989). Backpropagation applied to handwritten zip code recognition. Neural Comput. 1, 541–551. 10.1162/neco.1989.1.4.541

[B31] Lecun Y. Bottou L. Bengio Y. Haffner P. (1998). Gradient-based learning applied to document recognition. Proc. IEEE 86, 2278–2324. 10.1109/5.726791

[B32] Leow A. D. Yanovsky I. Parikshak N. Hua X. Lee S. Toga A. W.. (2009). Alzheimer's disease neuroimaging initiative: a one-year follow up study using tensor-based morphometry correlating degenerative rates, biomarkers and cognition. NeuroImage 45, 645–655. 10.1016/j.neuroimage.2009.01.004 19280686 PMC2696624

[B33] Lin C. Watson R. Ward H. Rydberg C. Witte R. Bernstein M. (2006). MP-RAGE compared to 3D IR SPGR for optimal T1 contrast and image quality in the brain at 3T. Int. Soc. Magn. Reson. Med. 14:981 Available online at: https://cds.ismrm.org/ismrm-2006/files/00981.pdf

[B34] Maaten L. V. D. Hinton G. (2008). Visualizing data using t-SNE. J. Mach. Learn. Res. 9, 2579–2605. Available online at: https://www.jmlr.org/papers/v9/vandermaaten08a.html

[B35] Magnin B. Mesrob L. Kinkingnéhun S. Pélégrini-Issac M. Colliot O. Sarazin M.. (2009). Support vector machine-based classification of Alzheimer's disease from whole-brain anatomical MRI. Neuroradiology 51, 73–83. 10.1007/s00234-008-0463-x 18846369

[B36] Malone I. B. Cash D. Ridgway G. R. MacManus D. G. Ourselin S. Fox N. C.. (2013). MIRIAD-Public release of a multiple time point Alzheimer's MR imaging dataset. NeuroImage 70, 33–36. 10.1016/j.neuroimage.2012.12.044 23274184 PMC3809512

[B37] Marcus D. S. Fotenos A. F. Csernansky J. G. Morris J. C. Buckner R. L. (2009). Open access series of imaging studies: longitudinal MRI data in nondemented and demented older adults. J. Cogn. Neurosci. 22, 2677–2684. 10.1162/jocn.2009.21407 19929323 PMC2895005

[B38] Marcus D. S. Wang T. H. Parker J. Csernansky J. G. Morris J. C. Buckner R. L. (2007). Open access series of imaging studies (oasis): cross-sectional MRI data in young, middle aged, nondemented, and demented older adults. J. Cogn. Neurosci. 19, 1498–1507. 10.1162/jocn.2007.19.9.1498 17714011

[B39] McCullagh P. (1984). Generalized linear models. Eur. J. Oper. Res. 16, 285–292. 10.1016/0377-2217(84)90282-0

[B40] Mehmood A. Maqsood M. Bashir M. Shuyuan Y. (2020). A deep Siamese convolution neural network for multi-class classification of Alzheimer disease. Brain Sci. 10:84. 10.3390/brainsci10020084 32033462 PMC7071616

[B41] Mueller S. G. Weiner M. W. Thal L. J. Petersen R. C. Jack C. R. Jagust W.. (2005). Ways toward an early diagnosis in Alzheimer's disease: the Alzheimer's disease neuroimaging initiative (ADNI). Alzheimer's Dement. 1, 55–66. 10.1016/j.jalz.2005.06.003 17476317 PMC1864941

[B42] Nair V. Hinton G. E. (2010). Rectified linear units improve restricted Boltzmann machines, in The 27th International Conference on Machine Learning (ICML 2010), eds Fürnkranz J. Joachims T. (Haifa: Omnipress), 807–814.

[B43] Pedregosa F. Varoquaux G. Gramfort A. Michel V. Thirion B. Grisel O. (2011). Scikit-learn: machine learning in python. J. Mach. Learn. Res. 12, 2825–2830.

[B44] Rieke J. Eitel F. Weygandt M. Haynes J.-D. Ritter K. (2018). Visualizing convolutional networks for MRI-based diagnosis of Alzheimer's disease. ArXiv e-prints. 10.1007/978-3-030-02628-8_3

[B45] Sharif Razavian A. Azizpour H. Sullivan J. Carlsson S. (2014). CNN features off-the-shelf: an astounding baseline for recognition, in 2014 IEEE Conference on Computer Vision and Pattern Recognition (CVPR) (Columbus, OH), 806–813. 10.1109/CVPRW.2014.131

[B46] Simonyan K. Zisserman A. (2014). Very deep convolutional networks for large-scale image recognition. arXiv. arXiv:1409.1556

[B47] Sørensen L. Igel C. Pai A. Balas I. Anker C. Lillholm M.. (2017). Differential diagnosis of mild cognitive impairment and Alzheimer's disease using structural MRI cortical thickness, hippocampal shape, hippocampal texture, and volumetry. NeuroImage 13, 470–482. 10.1016/j.nicl.2016.11.025 28119818 PMC5237821

[B48] Springenberg J. T. Dosovitskiy A. Brox T. Riedmiller M. (2014). Striving for simplicity: the all convolutional net. arXiv. arXiv:1412.6806.

[B49] Szegedy C. Liu W. Jia Y. Sermanet P. Reed S. Anguelov D. (2015). Going deeper with convolutions, in 2015 IEEE Conference on Computer Vision and Pattern Recognition (CVPR) (Boston, MA), 1–9. 10.1109/CVPR.2015.7298594

[B50] Thomann P. A. Kaiser E. Schönknecht P. Pantel J. Essig M. Schröder J. (2009). Association of total tau and phosphorylated tau 181 protein levels in cerebrospinal fluid with cerebral atrophy in mild cognitive impairment and Alzheimer disease. J. Psychiatry Neurosci. 34, 136–142. 19270764 PMC2647572

[B51] Thompson P. M. Hayashi K. M. de Zubicaray G. Janke A. L. Rose S. E. Semple J.. (2003). Dynamics of gray matter loss in Alzheimer's disease. J. Neurosci. 23, 994–1005. 10.1523/JNEUROSCI.23-03-00994.2003 12574429 PMC6741905

[B52] Tustison N. Avants B. (2013). Explicit b-spline regularization in diffeomorphic image registration. Front. Neuroinform. 7:39. 10.3389/fninf.2013.00039 24409140 PMC3870320

[B53] van der Walt S. Colbert S. C. Varoquaux G. (2011). The numpy array: a structure for efficient numerical computation. Comput. Sci. Eng. 13, 22–30. 10.1109/MCSE.2011.37

[B54] Wachinger C. Reuter M. (2016). Domain adaptation for Alzheimer's disease diagnostics. NeuroImage 139, 470–479. 10.1016/j.neuroimage.2016.05.053 27262241 PMC4983466

[B55] Weiler M. Agosta F. Canu E. Copetti M. Magnani G. Marcone A.. (2015). Following the spreading of brain structural changes in Alzheimer's disease: a longitudinal, multimodal MRI study. J. Alzheimer's Dis. 47, 995–1007. 10.3233/JAD-150196 26401778

[B56] Welford B. P. (1962). Note on a method for calculating corrected sums of squares and products. Technometrics 4, 419–420. 10.1080/00401706.1962.10490022

[B57] Wen J. Thibeau-Sutre E. Diaz-Melo M. Samper-González J. Routier A. Bottani S.. (2020). Convolutional neural networks for classification of Alzheimer's disease: overview and reproducible evaluation. Med. Image Anal. 2020:101694. 10.1016/j.media.2020.101694 32417716

[B58] Wyman B. T. Harvey D. J. Crawford K. Bernstein M. A. Carmichael O. Cole P. E.. (2013). Standardization of analysis sets for reporting results from ADNI MRI data. Alzheimer's Dement. 9, 332–337. 10.1016/j.jalz.2012.06.004 23110865 PMC3891834

[B59] Zeiler M. D. Fergus R. (2014). Visualizing and understanding convolutional networks, in European Conference on Computer Vision (ECCV) (Zurich: Springer), 818–833. 10.1007/978-3-319-10590-1_53

